# Detailed functional results after bladder-preserving surgery and high-dose-rate brachytherapy in pediatric bladder/prostate rhabdomyosarcoma

**DOI:** 10.1007/s00432-022-04209-5

**Published:** 2022-07-25

**Authors:** Verena Ellerkamp, Andreas Schmidt, Steven W. Warmann, Franziska Eckert, Juergen Schaefer, Frank Paulsen, Joerg Fuchs

**Affiliations:** 1 Department of Paediatric Surgery and Paediatric Urology, University Children’s Hospital, Eberhard Karls University Tuebingen, Hoppe-Seyler-Strasse 3, 72072 Tuebingen, Germany; 2 Department of Radiation Oncology, Medical University Vienna, Comprehensive Cancer Center Vienna, Vienna, Austria; 3 Department of Paediatric Radiology, University Hospital, Eberhard Karls University Tuebingen, Tuebingen, Germany; 4 Department of Radiation Oncology, University Hospital, Eberhard Karls University Tuebingen, Tuebingen, Germany

**Keywords:** Pediatrics, Rhabdomyosarcoma, Bladder reconstruction, Functional outcome, Urodynamics, Brachytherapy, Bladder sparing surgery, Bladder-preserving surgery

## Abstract

**Purpose:**

In pediatric bladder/prostate-rhabdomyosarcoma, the rate of bladder preservation after neoadjuvant chemotherapy is high, with an excellent oncological outcome. Information about functional urological long-term outcomes is rare.

**Methods:**

Data of all patients who had undergone bladder-preserving surgery with or without brachytherapy at our institution between 2009 and 2020 were analyzed retrospectively. Detailed urological function was assessed focusing on age-related continence, bladder capacity and urodynamic findings.

**Results:**

We identified 40 patients, median age at surgery of 27 months (range 9–191), and 32 patients additionally received postoperative high-dose-rate brachytherapy. The median follow-up was 32.5 months (range 6–125). The bladder capacity increased from median 66.7% (21.1–180) of expected bladder capacity related to age 3 months after surgery to 87.4% (58.1–181.8) 9 months after surgery. In the group of aged > 6-year-old, continence was 94% (83% with brachytherapy, 100% without brachytherapy). Erectile function was normal in 92% (90% with brachytherapy, 100% without brachytherapy). Bladder capacity was more than 65% expected bladder capacity related to age in 70% (60% with brachytherapy, 86% without brachytherapy). 65% of all patients need neither anticholinergic drugs nor low-dose antibiotics (63% with brachytherapy, 71% without brachytherapy).

**Conclusions:**

Bladder preservation with good functional outcome can be achieved in localized bladder/prostate-rhabdomyosarcoma. In selected cases, supportive brachytherapy additionally contributes to an improvement in the oncological outcome with calculable risks for bladder and erectile function. Careful urological aftercare should be a fixed priority after oncological follow-ups.

## Introduction

The overall annual incidence of rhabdomyosarcoma (RMS) in Europe is 5.4 cases per million (children < 15 years) (Martin-Giacalone et al. 2021). In 15–20%, RMS is located in the genitourinary system, most of which arises from the bladder/prostate region (BP-RMS) (Crist et al. 2001; Sultan et al. 2009; Arndt et al. 2004). Low risk localized embryonal BP-RMS has a 5-year event-free survival (5y-EFS) rate of 80%, while high-risk BP-RMS is associated with an 5y-EFS rate of only 65%. The gross amount of BP-RMS is not amendable to achieve primary complete excision at presentation (Rodeberg et al. 2011). Multimodal treatment concepts including radiotherapy and surgery are superior to chemotherapy alone in terms of 5y-EFS (Seitz et al. 2016). We recently showed that combining high-dose-rate brachytherapy (HDR-BT) with surgery after neoadjuvant chemotherapy results in a higher bladder preservation rate and improves the 5y-EFS compared to surgery alone (Schmidt et al. 2020). The oncologic outcome is comparable to that of low-dose-rate brachytherapy (LDR-BT) (Martelli et al. 2016) with all advantages compared to external radiotherapy (ERT). This study aimed to analyze the functional results of bladder-preserving surgery (BPS) with and without HDR-BT.

## Methods

The data of all patients with localized BP-RMS at our institution from 2009 to 2020 were analyzed retrospectively (ethical committee approval No. 353/2019BO2). All patients underwent risk-adapted neoadjuvant chemotherapy according to CWS protocols (Seitz et al. 2016; Koscielniak and Klingebiel 2014). Prior to surgical steps, a diagnostic cystoscopy was performed. The respective procedure was defined on the basis of the preoperative MRI and the intraoperative conditions and the patients were divided into two groups as described previously (Schmidt et al. 2020): bladder-preserving surgery (BPS) with HDR- BT (BPS + BT), BSP without BT (BPS). In case of a tumor extension beyond the prostate or above the trigone, a cystectomy was performed; these patients were excluded from this study. Additional BT was performed, if the following preconditions were fulfilled: Tumors were located at the level of or caudal of the bladder trigone; a R0 or R1 resection at the level of the bladder was aimed at; no requirement for resection of the urethral sphincter and preservation of at least half of the trigone. A symphysiotomy was performed for better surgical exploration. If necessary, bladder neck reconstruction was performed using lateral rotational flaps. In cases of infiltrated ureteral meatus, a transtrigonal ureteroneocystostomy or transverse ureteroureterostomy was performed. The urethra was splinted with a transurethral bladder catheter, into which one of the BT tubes was inserted. An additional suprapubic catheter was then inserted. BT tubes were placed in the former tumor location and around the prostate and were removed immediately after the last BT session. The details of the HDR-BT protocol have been described previously (Fuchs et al. 2016). Briefly summarized, postoperatively a three-dimensional brachytherapy planning is performed on base of planning CT and postoperative MRI scan (Brachyvision, Varian, Medical Systems, Haan, Germany). The clinical target volume (CTV) encompasses the tumor site underneath the trigonum including the prostate. The real dose distribution depends on the localization of the tubes. Treatment planning goal is an optimal coverage of the CTV (95% of the defined CTV with at least 95% of the prescribed dose) with avoidance of high radiation doses at the rectum, growth plates, gonads, urethra and bladder. The prescription constraints for organs at risk are a process of work in progress considering the growing clinical experience in this rare entity and the individuality of each patient. HDR-BT was applied with a ^192^Ir HDR source in fractions of 3 Gy, twice daily, with weekend rest periods until a total dosage of 36 Gy is reached. Patients remained ventilated during the hole period to avoid BT-tube dislocation.

Voiding cystourethrography (VCUG) was performed two weeks postoperatively. Complications were recorded and classified (Clavien–Dindo) (Dindo et al. 2004). Follow-up oncological examinations (MRI, genitoscopy) were performed as described previously (Schmidt et al. 2020). The urological outcome was assessed as follows: bladder capacity (BC), micturition frequency and volume, age-related continence, defined as no need for diapers at day, uroflowmetry, videourodynamic studies (VUD) if any anomalies of the previous, morphological/functional changes of the upper urinary tract, medication (antibiotic prophylaxis, anticholinergic therapy), and erectile function (EF).

As for infants and young children, measurement of maximal BC is difficult to achieve, but BC under anesthesia was measured using regular MRI. BC was calculated as 4/3*π*a*b*c (a, b, and c are the half-axes). The expected BC related to age (EBCA) was calculated according to the formula of Hjalmås [(Age[years] + 1)*30]; BC of 65% or more of EBCA was assessed as normal. If micturition protocols were possible, or VUD was performed, these values were compared to the calculated BC on MRI.

VUD was performed according to recent recommendations of the ICCS (Austin et al. 2016). A ‘normal’ value for compliance in adults has not been validated but values > 20 ml/cmH2O are generally accepted as normal, corresponding to about 5% of normal bladder capacity. Following this definition, in children, the expected age-appropriate bladder compliance (EABCom) was calculated as 5% age-related bladder capacity (Lapointe and Barrieras 2005). If the compliance was below 65% of the age-related value, it was considered insufficient. Additionally, to characterize the reservoir properties, BC at 20 cmH_2_O (truly safe) and 30 cmH_2_O (borderline value) baseline detrusor pressure was registered.

### Statistics

Data were analyzed with SPSS Statistics for Windows version 26 (IBM software). The decision for parametric or non-parametric tests was made after the Shapiro–Wilk test. Non-parametric data are expressed as medians and (ranges). Comparisons of group data were performed using the Mann–Whitney *U* tests (ordinal scale) or Chi-square test (nominal scale). A *p* value of ≤ 5% was considered statistically significant. The 5y-OS and -EFS were calculated using Kaplan–Meier tests.

## Results

During this period, 40 patients underwent BPS (Table 1). The median age at surgery was 27 months (9–191). BT was performed in 30 patients, and additionally in 2 patients with relapse (Table 1). The median follow-up was 31.5 months (6–125). In one patient, the family initially refused the recommended local therapy after neoadjuvant chemotherapy; 21 months after the initial diagnosis, the tumor relapsed. Salvage chemotherapy was administered followed by BPS + BT. The patient died 23 months later from local relapse and distant metastases.

**Table 1:** Oncological aspects, surgical details and complications

	BSP with BT *N* = 30	BSP without BT *N* = 10	*p* value
Demographics			
Age at surgery [months]	25.5 (9–191)	38,5 (12–59)	0.770
Length of FU [months]	32.5 (6–125)	40 (13–125)	0.363
Interval diagnosis – local therapy [months]	5 (0–32)	4,5 (3–29)	0.866
Gender male	29	6	
Tumor size			
< 5 cm	16	7	0.356
> 5 cm	14	3	
Cranio-caudal median diameter [mm], (range)	41.5 (23–140)	38.5 (9–77)	0.325
Tumor localization			
Bladder	9	7	0.071
Bladder/prostate	18	3	
Prostate	3	0	
Risk group			
Standard risk	15	7	0.271
High risk	15	3	
Histology			
Embryonal RMS	20	7	0.336
Botryoid subtype	9	3	
Focal anaplasia	1	0	
Surgery			
Resection trigonum, total	10	6	
Partial trigone resection	0	3	
Ureteral reimplantation patients/sides	5/7	8/10	
Ureteroureterostomy	1	1	
Prostatectomy, total	1	3	
Prostatectomy, 50%	9	1	
Bladder neck reconstruction	20	4	
Biopsy only	2	0	
Complications			
Urinary leakage (Clavien–Dindo grade I)	8	3	
Rectourethral fistula (Clavien–Dindo grade IIIb)	1	0	
Fibrotic ureter (Clavien–Dindo grade IIIb)	1	0	
Radiation urethritis (Clavien–Dindo grade I)	3	0	
Resection status			
R0 (microscopically complete)	17	10	0.40
R1 (macroscopically complete)	12	0	
R2 (macroscopically incomplete)	2	0	
Outcome			
5y-OS (95% CI)	94.7% (84.7–100)	100%	0.491
5y-EFS (95% CI)	74.4%** (56–92.8)	65.6%*** (33.5–97.7)	0.724

*BT* brachytherapy, *SR* standard risk, *HR* high risk, *EFS* event-free survival, *OS* overall survival, *CI* confidence interval

**6 relapses 3, 6, 7,8,10 and 14 months after surgery and BT

***3 relapses 4, 14, and 17 months after surgery microscopic residuals (R1)

The 5y-OS, and -EFS, was 95.8% (95% CI 87,76–100), and 71.3% (95% CI 54.6–87.96). Seven patients with relapses underwent microscopic complete resection (R0), while two had microscopic residuals (R1).

Among the patients with relapse, half of them had a low-risk classification, while the other part was classified as high-risk.

Urinary leakage from the reconstructed bladder or urethra was the most common complication (Table 1). Conservative treatment with catheter retention for a median period of 65 days (25–147) led to spontaneous closure in all cases. In patients without leakage, suprapubic catheters were removed after a median of 23 days (25–147). The wide range in the latter was an effect of 5 patients (4 of the BPS + BT group) with prolonged median bladder training of 171 days (67–251) compared to patients with normal median bladder training of 4 days (0–20). One rectourethral fistula required a protective colostomy and stenting of the urethra. The colostomy was closed after spontaneous occlusion of the fistula. A fibrotic distal ureter resulted in secondary transverse ureteroureterostomy. In three patients, urethritis was diagnosed during follow-up examinations.

In seven patients, persistent VUR was diagnosed, and five of these had no ureteral surgery. Of these, two needed anticholinergic medication, another one was medicated with low-dose long-term antibiotics, and three more needed both. Three patients had signs of ureteral obstruction (megaureter and hydronephrosis), two after ureteral surgery, one without. One patient with congenital ureteral pelvic junction obstruction and dismembered pyeloplasty before RMS diagnosis developed stage 3 chronic kidney disease (CKD). No other patient showed signs of CKD, elevated creatine, or cystatin C.

For urological evaluation, three patients were excluded (one death, one cystectomy due to relapse, one with incomplete urological follow-up). Two other patients in the BPS group with relapse were treated with BPS + BT and further assigned to the BPS + BT group (Table 2). At the time of last follow-up, 51% patients were < 6 years. A 12-year-old boy suffered from weak.

**Table 2:** Age-related urological details

	BPS + BT (*n* = 30)						BPS (*n* = 7)					
	0–5 years (*n* = 16, 1 girl)			> 6 years (*n* = 14, 1 girl)			0–5 years (*n* = 2)			> 6 years (*n* = 5, 3 girls)		
Median age at follow-up [months]	75.5 (23–194)											
	72.5 (23–194)						82 (51–158)					
	57 (23–118)			102 (74–194)			52 (51–53)			86 (79–158)		
Median follow-up [months]	22 (6–104)			52 (6–125)			36 (13–39)			60 (30–125)		
	n	%	95%CI	n	%	95%CI	n	%	95%CI	n	%	95%CI
Erectile dysfunction	1	6.7	0.0–19	2	15.4	0–35	0	0	0–0	0	0	0–0
Enuresis nocturna	7	43.8	19–68	4*	28.6	41–52	2	100	100	0	0	0–0
Incontinence daytime	6	37.5	14–61	3^**#**^	21.4	0–43	1	50	0–100	0	0	0–0
*Urinary dribbling*	3	18.8	0–38	2^***§***^	14.3	0–33	0	0	0	0	0	0–0
*Urge*	1	6.3	0–18	1	7.1	0–21	0	0	0	1	20	0–55
Protections												
At night	2	12.5	0–29	3	21.4	0–43	1	50	0–100	0	0	0–0
Day and night	7	43.8	19–68	1	7.1	0–21	1	50	0–100	0	0	0–0
Percent of age-adjusted bladder capacity												
< 65%	7	43.8	19–68	3	21.4	0–43	0	0	0	1	20	0–55
≥ 65%	9	56.3	32–80	11	78.6	57–100	2	100	100	4	80	45–100
Medication												
Anticholinergica	6	37.5	14–61	4	8.6	49–52	1	50	0–100	1	20	0–55
Low-dose antibiotics	4	25.0	4–46	1	7.1	0–21	1	50	0–100	0	0	0–0
None	9	56.0	32–80	10	71.4	48–95	1	50	0–100	4	80	45–100

Confidence interval as normal approximation to the binominal calculation

*In 1 pt. 6–7 years old

^#^In two 6 years old pts., in one 12 years old pt

^*§*^In two 6 years old pts

erections and hypogonadism (hormonal replacement); in two other patients (4-year-old and 7-year-old boys) erectile dysfunction (ED) was assumed (parental observation). In the age group > 6 years, 15 out of 18 patients (83%) were continent. Twelve patients needed anticholinergic medication, 7 patients needed antibiotic prophylaxis, and 24 patients needed no medication at all. Uroflowmetry was possible in 26 patients, all of whom had normal flow curves and adequate maximum flow rate.

**Fig. 1:**
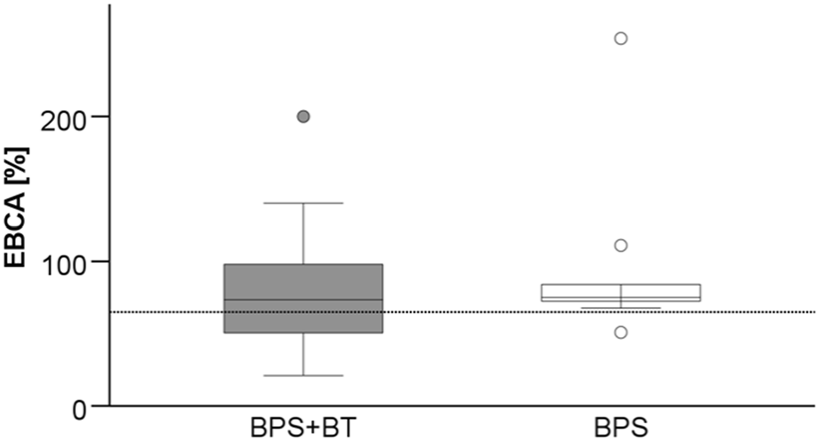
BC in relation to EBCA (BC/(30 × [age + 1])*100) at last follow-up. The dotted line indicates the limit below which children are considered to have decreased bladder capacity compared to estimated bladder capacity for their age (65% EBCA).) There was no significant difference between the groups (BPS + BT: 72.8% (21–200) vs. BPS: 78.7% (50.9–254.1); *p* = 0.601, Mann–Whitney U test)

**Fig. 2:**
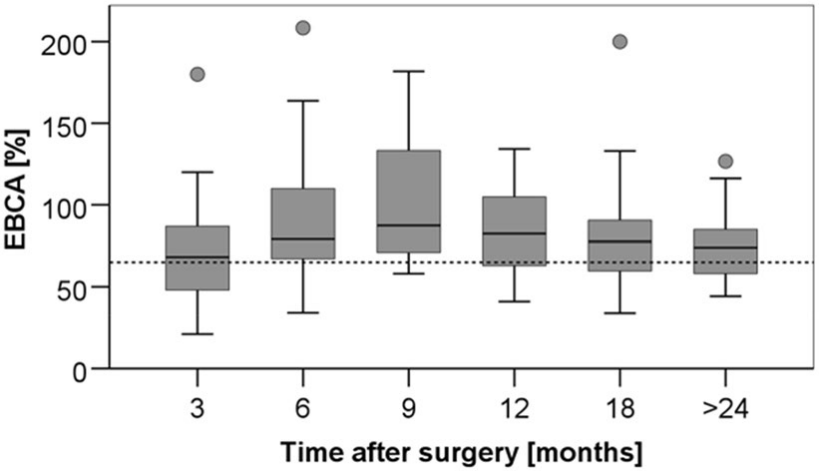
Median rate of BC related to EBCA. The dotted line is the limit below which children are considered to have decreased bladder capacity compared to estimated bladder capacity for their age (65% EBCA). There was an initial increase from median 66.7% (21.05–180) after 3 months to 79.1% (34.1–254.1) after 6 months, and to 87.4% (58.1–181.8) 9 months after surgery. Then it decreased again to 82.4% (41.0–134.2) 12 months after surgery and to 79.6% (36.8–200.0) 18 months and to 74.0% (44.2–126.7) > 24 months after surgery. The variation of the median values was not significant (*p* = 0.41, Kruskal–Wallis test). There was no significant difference between the two groups (graphs not shown)

At a median follow-up of 32.5 months (6–125), the median BC averaged 74% (range 21–254%) of EBCA (Fig. 1). Over time, there was an initial increase, followed by a decrease in BC after surgery (Fig. 2). Comparing the median BC gained by MRI with those of urodynamics, or micturition protocols in patients in whom both values were available (*n* = 34), no significant difference was found (MRI 73.3% EBCA, (36.8–181.8) vs. urodynamics/micturition protocols 79.1% EBCA, (21.1–227.2)).

**Fig. 3:**
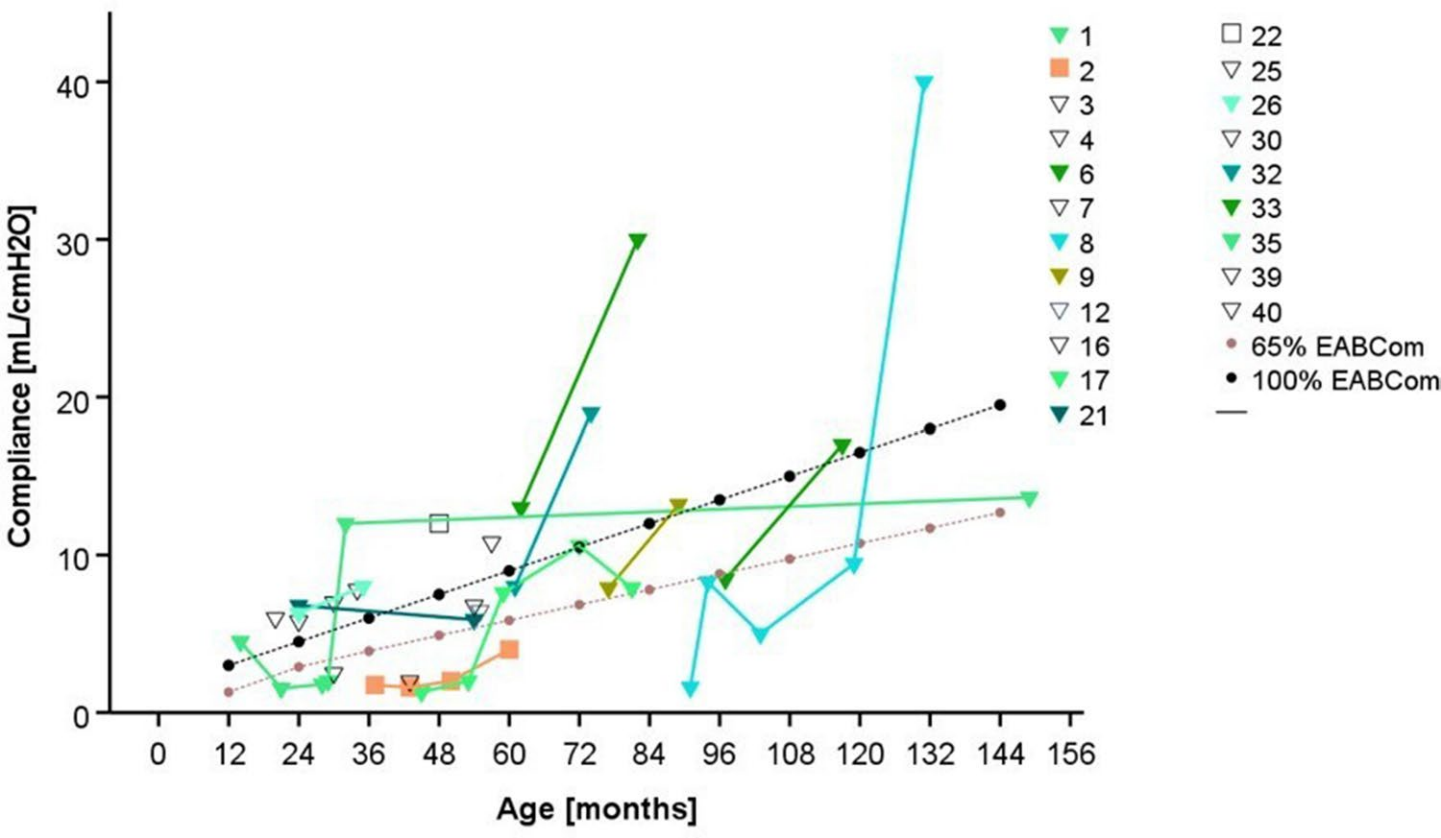
Compliance of patients with UD. Black line with dots—100% EBACom, gray line with dots—65% EABCom. Triangles show patients of BPS + BT group, Squares show patients of BPS group. Most patients improve at time with anticholinergic drugs. Deterioration of Pt. 35 was due to lack of drug adherence

Due to different combinations of clinical findings, in 21 children VUD was performed, in 11 of them more than one. Nine children underwent uneventful examination. Five children had elevated maximum detrusor pressure (PdetMax; three patients 36–40 cmH2O, two patients > 40 cmH2O). Six children demonstrated an initial compliance < 65% of EABCom and reduced BC at pressures of 20 and 30 cmH2O. VUR was detected in five of these cases. With anticholinergic drugs, compliance improved in all patients (Fig. 3) but decreased again in one patient due to poor drug adherence. Detrusor pressures improved in four patients (two patients PdetMax 31–35 cmH2O, one patient 25–30 H2O, one patient < 20 cmH2O), and remained elevated in the patient with poor drug adherence. In two patients, follow-up VUD is pending.

## Discussion

Optimization of multimodal treatment protocols led to an increase in OS rates for high-risk BP-RMS to more than 80% (Mandeville 2019; Saltzman and Cost 2018). Neoadjuvant chemotherapy is the backbone of all therapeutic strategies. A crucial aspect of multimodal treatment is the type of RT. Focusing on the potential toxicities of radiotherapy (bladder fibrosis, bowel injury, pelvic bone deformities, hypogonadism), attempts to reduce the dose to surrounding tissues are important (Cotter et al. 2011). Based on the favorable results of BT in vulval/vaginal RMS since the early 1970s in France (Flamant et al. 1990), a first report proved the advantages of this technique in combination with BPS in patients with BP-RMS (Martelli et al. 2009). The main advantage of BT is reduced irradiated volumes with a corresponding lower risk of late toxicity (Naghavi et al. 2017). Since then it has been generally accepted that BT, as well as proton therapy, is superior to photon radiotherapy in terms of doses to the surrounding tissues (Heinzelmann et al. 2011). The oncological outcome is convincing; in a large prospective cohort, the combination of BPS with BT facilitated a 5y-EFS and -OS rate of 84%, respectively, 91% (Chargari et al. 2017; Schmidt et al. 2020; Zakem et al. 2022). In the recent update of our findings, we had to add one relapse-related death. Because of the special circumstances with initial denial of local therapy, it can be assumed that this death could have been avoided. Simultaneously, this case emphasizes the importance of timely local therapy. Other studies with longer postoperative follow-up times but smaller study cohorts reported 100% EFS rates after BT in BP-RMS patients (Lobo et al. 2022; Stenman et al. 2022).

A direct comparison of oncological outcomes is compromised, with either no information about the IRS stage being given (Lobo et al. 2022) or incomplete information about tumor diameters (Stenman et al. 2022). This emphasizes the importance of accurately describing key oncologic data, even when the focus is on functional outcomes. Only one study provided a full description of oncological details, with a group size comparable to our cohort (Martelli et al. 2016) and an oncological outcome comparable to ours. In contrast to our approach, these authors used LDR-BT.

The most common complication in our patients was postoperative leakage of the reconstructed urethra or bladder, reflecting the results of other groups. Three cases of radiation urethritis in our group found no corresponding cases in follow-up studies of other groups, but urethral stricture as a late complication has been described (Martelli et al. 2016).

In the adult section, urinary incontinence rates vary from 0 to 19% (Leapman et al. 2016). In pediatric patients, objective urological outcomes are more difficult to gather due to age; micturition protocols, voiding frequencies or measurements of maximum voided volumes are difficult to obtain in the diaper age. Furthermore, families, surgeons and oncologists, have primarily focused on oncological outcomes. Additionally, very often, only short-term follow-up is performed by the centers of local therapy, and many patients are followed-up by their oncologists close to home. For this reason, some authors refer to questionnaires to assess the results after longer follow-up periods (Arndt et al. 2004; Frees et al. 2016; Stenman et al. 2022), or even only describe anamnestic findings (Chargari et al. 2017; Indelicato et al. 2020) (Table 3).

**Table 3:** Review of oncological and urological outcome

Study	No (male), Urological evaluated	Surgery, No	Radiation, No (dosage)	Median follow-up [months]	Median age at FU	Survival and relapse	Normal voiding function	Normal erectile function/ Hormonal replacement	Outcome measurements, No
Recent Study	40 (36), 37	BPS, 38	HDR-BT, 32 (30–36 Gy)	37	6 years	96% OS 70% EFS 9 relapses 1 death	83% ****	92% 2.7%	Bladder capacity, 37 Residual urinary volume, 30 Uroflowmetry, 26 Urodynamics, 21
Stenman et al. (2022)	10 (7), 10	BPS, 5	HDR-BT, 10 (39–42 Gy)	62	No data	100% OS 90% EFS	100%	100% 4.3%	Bladder capacity, 8 Uroflowmetry, 8 Residual urinary volume, 8 Urodynamics, 1 Questionnaire, 8
Lobo et al. (2022)	13 (10), 13	BPS, 2 EP, 4	HDR-BT, 13 (27.5 Gy)	42	6 years	No deaths No relapse	62%	No data	Bladder capacity, 13 Uroflowmetry, 13 Urodynamics, 2
Indelicato (2020)	31	BPS, 8	PB, 31, (36–50.4 Gy)	48	No data	84% OS 80% EFS	89%	No data	Anamnestic, 31
Chargari et al. (2017)	32 (26), 7/14*	BPS, 29	LDR/PDR-BT, 32 (60 Gy)	20	> 6	91% OS 84% EFS	71%	100%	Anamnestic, 7; 14*
Frees et al. (2016)	13 (13), 13	BPS, 13	RT or BT, 3 (no data)	152	20 year	No data	No data	24%	Questionnaire, 13
Martelli et al. (2016)	27 (27), 22	BPS, 27	LDR-BT, 27 (60 Gy (20 Gy + 45 Gy ERT))	120	13 years	3 deaths	55%	100% *‡*	Urodynamics, 11 Questionnaire, 22
Martelli et al. (2009)	26 (26), 22	BPS, 26	LDR-BT, 26 (60 Gy)	48		2 deaths	82% **	100% *‡* 25% *‡‡*	Anamnestic, 22 Urodynamics, 1
Arndt et al. (2004)	55	BPS, 55	XRT, 55, 41–59.4 Gy	No data	No data	82% OS 77% EFS	65% ***	No data	Urodynamics, 1 Questionnaires, 23

*EP* endoscopic polypectomy, *ERT* external radiotherapy, *HDR* high-dose-rate, *LDR* low-dose-rate, *PDR* pulse-dose-rate, *RT* radiotherapy, PB proton beam

*7 pts. aged > 6 years were evaluated concerning continence, 14 pts. aged > 4 were evaluated concerning erections

**Dribbling, incontinence, hydronephrosis, enuresis before the age of 10 years considered “normal”; ***of 11 pts. > 6 years

****Of 18 pts. > 6 years

^‡^Pubertal / adolescent pts

^‡‡^Normal erections observed by parents in 5 of 20 pts

As during oncological follow-up, regular MRI in narcosis is performed, BC can be easily calculated in a relaxed child noninvasively. With this method, it was possible to objectify BC related to EBCA and correlate it to the postoperative period. Our data showed an increase in bladder capacity during the first 9 months after BPS + BT and a slight decrease during the following 15 months. At a median follow-up of 2.5 years and a median age of 5.8 years, our patients had a bladder capacity of 74% of EBCA without a significant difference between the groups. Only one article described BC after BT, these authors found a median BC of 75% EBCA at a median age of 6 years in two cases after partial cystectomy with BT compared to 96% EBCA after endoscopic polypectomy with BT (Lobo et al. 2022).

Furthermore, the assessment of compliance in this age group, if urodynamic tests are performed at all, is limited by the lack of normal values. A reduced compliance value denotes an increase in bladder wall stiffness and is a risk factor for upper tract damage. In our cohort, compliance values were mostly well below 10 ml/cmH2O. However, with regard to EBACom, the majority of the measured values were within the normal range and improvement due to anticholinergic drugs could be made visible. In other studies, urodynamic testing was rarely performed and urodynamic findings were not provided in detail (Arndt et al. 2004; Lobo et al. 2022; Martelli et al. 2009; Stenman et al. 2022), except in one study (Martelli et al. 2016).

In adults after BT without ED before therapy, ED is described in up to 21% of adults after BT. There was no significant difference between HDR- and LDR-BT in long-time sequelae in the elderly (Johansson et al. 2021). In the early follow-up of pediatric patients, EF can only be obtained by parental observation, which entails a certain bias. Interestingly, a study of adult long-term survivors of pediatric BP-RMS described ED in 100% after radical surgery with adjuvant radio-/ or brachytherapy and in 70% after radical surgery only. This is the only study on adults with questionnaires but also deals with low number of patients who were treated with surgery and radio-/brachytherapy (Frees et al. 2016). Other studies reported approximately 100% normal EF in low numbers of adolescent or adult patients, controversially (Chargari et al. 2017; Martelli et al. 2009, 2016; Stenman et al. 2022). With the awareness of possible later impairment of EF, we learned that well-informed parents were able to observe the EF of their sons fairly well. However, this may only be a hint of later outcomes in this respect. In the herein reported patient cohort, three patients had ED, representing 10% of the BPS + BT group. Testosterone deficiency has been reported in adults with prostate cancer after radiation therapy even without androgen deprivation therapy. In adult patients after permanent interstitial BT for localized prostate cancer, initially decreased testosterone levels recovered at 18 months after BT (Taniguchi et al. 2019). Cases of hypogonadism in adolescents after BT in childhood have been described rarely (Stenman et al. 2022). In our cohort, one boy needs hormonal replacement therapy. As most of our patients were still in a prepubertal age, further follow-up studies will be conducted.

In summary, this recent study is the first with a detailed analysis of functional urological outcomes according to age groups after BPS and HDR-BT and the largest series with available urodynamic studies. The reported rates of normal bladder function after multimodal bladder-preserving therapies is differing in the literature from 55 to 100% (Arndt et al. 2004; Lobo et al. 2022; Chargari et al. 2017; Frees et al. 2016; Indelicato et al. 2020; Martelli et al. 2009, 2016; Stenman et al. 2022). Our cohort with 83% normal bladder function is well in range with these. Many studies, including small patient cohorts (Lobo et al. 2022), are restricted by their ambiguous definition of normal bladder function (Arndt et al. 2004) and do not always describe surgical details since the primary focus is still on the oncological outcome. Current research also suggests, that problems of the lower urinary tract and/or ED may increase with a longer median follow-up (Frees et al. 2016; Castagnetti et al. 2019). Our study included 40 patients with BPS, 32 of whom received additional BT. Similar to other studies, our observation period was still too short to allow truly representative conclusions to be drawn, as more than half of the children were younger than 6 years at their last follow-up. Another limitation of our study is its retrospective nature.

In conclusion, short-term observations of urologic outcomes in patients with BP-RMS are encouraging, and long-term analyses are desirable. The urological outcome should be monitored as closely as the oncologic outcome to reflect a realistic overall impression in patients with BP-RMS.

## Data Availability

The datasets generated during and/or analyzed during the current study are available from the corresponding author on reasonable request.
